# Investigating the impact of fuel price management on public transport use a case study of public road transport in East Azerbaijan Province Islamic Republic of Iran 

**Published:** 2019-08

**Authors:** Mirreza Fatemi, Orujali Alizadeh

**Affiliations:** ^*a*^Road Safety Commission, East-Azerbaijan Road Maintenance and Transportation Organization.

## Abstract

In today's busy world, the use of the public fleet is preferable to the use of personal rides. Public transportation is safer and more economical and it has environmental benefits too. Therefore, governments have always been policy-making to encourage people to use the public transportation.

On the other hand the underdevelopment of urban and road infrastructure has been problematic in most societies. This article focuses on the impact of fuel price management on public transportation. Statistics of passenger transport during 2009 to 2019 by public passenger transport indicate that on average, about 10 percent of the number of passengers is reduced each year .This despite the fact that the number of personal rides is increasing every year and the number nationwide has grown from 10 million in 2009 to about 24 million in 2019.

Fuel price management is one of the most important factors influencing people's willingness to use the public fleet instead of personal cars. A look at fuel prices statistics, especially gasoline, shows that price growth has not been commensurate with the country's inflation rate and has seen a slight increase. Given that the quality and quantity of public road transport has improved over the years, as a result, the tendency to use a personal car for intercity travel has increased and road public transport passengers have declined. In other words, price management has not had a proper and targeted impact on public transport.

**Keywords:**

Public transport, Fuel price management, Personal rides

